# GuiErBai: a potent inhibitor, exhibiting broadly antitumor effect against cervical cancer *in vitro* and *in vivo*

**DOI:** 10.3389/fphar.2024.1296588

**Published:** 2024-06-10

**Authors:** Hong-en Qin, Lei Peng, Yuan-cui Xu, Zi-xiong Zhang, Ren-fu Tian, Zhong-xian Wan, Dao-jing Pu, Hong-chun Li, Fei Wu, Liangdong Zheng, Xian-shun Xu

**Affiliations:** Department of Medicine, The Central Hospital of Enshi Tujia and Miao Autonomous Prefecture, Enshi, Hubei, China

**Keywords:** cervical cancer, antitumor effect, Chinese traditional medicine, Chinese herbal medicine, podophyllotoxin

## Abstract

**Introduction:** Cervical cancer (CC) ranks as the fourth most prevalent malignant tumor among women worldwide, and is the fourth leading cause of cancer-related mortality. GuiErBai (GEB), a compound preparation developed by our research team, is derived from the ancient Chinese medicine of the Miao nationality and is comprised of podophyllotoxin (PTOX), imperatorin, isoimperatorin, and *A. dahurica* alkaloids. These individual components have demonstrated notable efficacy in tumor treatment. However, the specific anti-tumor effect of the compound Chinese medicine GEB in the context of CC has yet to be validated.

**Methods:** HeLa and SiHa cell lines were utilized for *in vitro* experiments and treated with 5 mg/mL and 10 mg/mL GEB concentrations, respectively. The cell cycle changes after GEB treatment were assessed using flow cytometry. Transmission electron microscopy was employed to observe autophagic bodies and apoptotic bodies, while MDC staining evaluated the occurrence of autophagy. CCK-8 was used to observe the effect of GEB on cell proliferation, and Transwell assays assessed cell migration and invasion. Western blotting detected cell cycle and apoptosis-related protein expression, along with the expression level of autophagy-related protein LC3I/II. Changes in ROS and mitochondrial membrane potential in cervical cancer cells following GEB treatment were determined using ROS detection and mitochondrial membrane potential detection kits. For the *in vivo* experiment, a nude mouse model of cervical cancer transplantation based on HeLa cells was established. Experimental animals were divided into negative control, positive control, high-dose GEB (10 mg/mL), and low-dose GEB (5 mg/mL) groups.

**Results:** In HeLa and SiHa cell lines, the G0/G1 phase of tumor cells significantly decreased (*p* < 0.001), while the G2/M phase increased notably (*p* < 0.001) following various GEB treatments. Electron microscopy showed GEB promoted apoptotic body and autophagosome formation in both cell lines. Compared to untreated HeLa and SiHa cells, GEB-treated cells exhibited significantly reduced caspase3 protein expression, and substantially increased autophagy-related protein LC3I/II expression. GEB treatment significantly reduced migration and invasion capabilities in both cell lines (*p* < 0.001), while ROS content and mitochondrial membrane potential were significantly elevated (*p* < 0.001). GEB effectively inhibited cervical cancer cell proliferation, with the optimal concentration being 10 mg/mL. A successful nude mouse model of cervical cancer transplantation was established using HeLa cells. Post-GEB treatment, the tumor volume and weight in nude mice significantly decreased (*p* < 0.001), with diminished expression of CD34, VEGF, and caspase3 proteins in tumor tissues.

**Discussion:** GEB exhibits a robust antitumor effect against cervical cancer, both *in vitro* and *in vivo*, in a concentration-dependent manner, by regulating autophagy and apoptosis of tumor cells.

## Highlights

• Cervical cancer is a common malignant tumor among women.
• Podophyllum is the rhizome of Dysosma versipellis (Hance) M. Cheng.
• GuiErBai (GEB) is composed of Podophyllum, D. dasycarps, and A. dahurica.
• GEB exhibits a broadly anti-tumor effect against cervical cancer.

## 1 Introduction

Cervical cancer (CC) ranks as the fourth most frequent malignant tumor and the fourth leading cause of cancer-associated mortality among women globally. As the predominant gynecological malignancy within the reproductive tract, CC affects approximately 13 per 100,000 individuals worldwide, with seven deaths attributable to the disease (Sung et al., 2021; Arbyn et al., 2020). CC is the most prevalent gynecological malignancy in the reproductive tract (Sung et al., 2021). In 2020, there were approximately 604,127 reported cases of cervical cancer globally, resulting in 341,831 deaths. This disease affects about 13 out of every 100,000 individuals worldwide, with seven deaths attributed to it (Singh et al., 2023). The average age of CC onset continues to decline, with the primary peak occurring between 45 and 49 years old. Moreover, the majority of CC patients present with locally advanced stages at hospital admission, specifically between Phase IB2 and IVA, as per the International Federation of Gynecology and Obstetrics (FIGO) classification (He WQ and Li C, 2021). Concurrent chemoradiotherapy is the standard-of-care treatment for locally advanced cervical cancer, which includes patients with stage IB3 to IVA disease, and it is effective for many patients; however, cervical cancer-related mortality remains high (Mayadev et al., 2021). Although targeted therapy and immunotherapy have recently advanced, their limited stability, high costs, and accompanying adverse reactions constrain widespread implementation (Ferrall et al., 2021). Consequently, conventional approaches, such as surgery, radiotherapy, and chemotherapy, remain prevalent in clinical practice, despite causing patients considerable physical trauma (Marnitz et al., 2020; Bhatla et al., 2021). Thus, researchers continue to explore novel treatments for advanced CC (Pal et al., 2022).

Continuous HPV infection is the cause of the majority of CC cases, and certain HPV subtypes such as E6*I and E6 have been associated with resistance to standard chemoradiotherapy (Ruiz et al., 2021). Previous scholars have suggested routine HPV testing for primary cervical cancer before treatment, to determine which patients may benefit from more aggressive precision-driven treatment (Ruiz et al., 2021). Over one-third of advanced CC patients fail chemoradiotherapy, and their resistance is closely related to the activation of inflammatory pathways and tumor infiltration of bone marrow-derived immune cells, especially macrophages (Floberg et al., 2021). The tumor microenvironment (TME) is a complex system composed of tumor cells, stromal cells, and immune cells, and it also plays an important role in the progression and resistance of CC. Disruption of helper T cells (Th) in the TME is an important mechanism leading to immune escape of tumors (De Nola et al., 2021). High cell density accumulation, extracellular matrix (ECM), and fluid shear stress between the ECM and surrounding disordered arteries in the TME can hinder the entry of chemotherapeutic drugs such as cisplatin into cancer cells, leading to resistance of cervical cancer (Bhattacharjee et al., 2022). In addition, cell-cell interactions in the TME can also induce resistance in cervical cells (Khalaf et al., 2021). For example, carcinoma-associated fibroblasts (CAFs) can induce tumor resistance through the secretion of chemokines or growth factors (IL-6, IL-8, IL-11, IGF-1, and TGF-β) (Mao et al., 2021). The use of natural and synthetic compounds to block or target the NF-κB signaling pathway, PI3K pathway, etc., is also a direction for the development of cancer therapy (Rashmi et al., 2020). Exploring targeted drugs for immune and inflammatory regulation pathways in CC is a focus of global scholars.

Traditional Chinese medicine (TCM) is an independent medical system that places great emphasis on holistic regulation and personalized interventions tailored to the unique characteristics of each patient (Wang et al., 2021). The effectiveness of TCM compounds and their active components in managing cervical cancer has been supported by modern pharmacology (Wang et al., 2021; Wang et al., 2021). Podophyllum, known as JiangBianYiWanShui among the Miao population in China, is a medicinal material derived from the rhizome of the Berberidaceae plant (Kalam et al., 2021). Podophylloideae includes Sinopodophyllum, Diphylleia, and Dysosma. In China, Podophyllum mainly comes from the rhizome of Dysoma versipellis (Hance) M. Cheng (D. versipellis), a plant of the Berberidaceae family. As a rare TCM, podophyllum has demonstrated significant efficacy in treating condyloma acuminatum induced by the human papillomavirus (HPV) (Wollina, 2003; Maleš et al., 2019). GuiErBai (GEB) is composed of Podophyllum*, Dictamnus dasycarps Turcz. (D. dasycarps), and Angelica dahurica (Fisch.ex Hoffm.) Benth. et Hook.f. (A. dahurica)*, and was originally formulated by our team. The main effective molecules in GEB include podophyllotoxin (PTOX), imperatorin, isoimperatorin, and A. dahurica alkaloids. Individual components such as PTOX (Guo and Jiang, 2021), imperatorin (Nasser et al., 2019), isoimperatorin (Kim et al., 2022), and A. dahurica alkaloid (Wang et al., 2018) have exhibited significant anti-tumor properties. However, the specific impact of the GEB compound in cervical cancer remains to be verified.

The current study aims to evaluate the anti-tumor properties of different concentrations of GEB on CC in both *in vitro* and *in vivo* settings. Developed independently by our research team, GEB is a Chinese medicine formula, and this study marks the inaugural evidence-based investigation of its effectiveness in treating CC.

## 2 Materials and methods

### 2.1 GEB preparation

*D. versipellis* (batch number: 20160702, place of origin: Enshi, China), *D. dasycarps* (batch number: 20160301, place of origin: Jiangsu), and *A. dahurica* (batch number: B707071-01, place of origin: Anhui) were acquired from Hubei Jurui Traditional Chinese Medicine Slices Co., Ltd. Following the prescribed proportions, the ingredients were extracted with water or ethanol. The extracts were refined using the optimal extraction process and the refining methods were compared, including alcohol precipitation, chitosan technique, and membrane separation method. The investigation focused on identifying the best refining approach and optimal process parameters, using the retention rate of the primary active components and solid retention rate as indicators (Gao et al., 2021). Consequently, the optimal GEB preparation was developed.

Preparation process: According to the prescription ratio, weigh the medicinal materials, use 80% ethanol for reflux extraction twice, with 9 times the amount for the first extraction and 7 times the amount for the second extraction. Extraction time is 60 min at a temperature of 85°C. Combine the extracts, filter, centrifuge (11,269.4 × *g*), recover the ethanol to obtain a dry extract (relative density 1.25-1.30, 60°C), vacuum drying (0.08 mP, 60°C), pulverize (pass through a No. Five sieve), seal, dry, and store for later use.

### 2.2 Cell culture and experiment *in vitro*

HeLa cells were chosen to represent adenocarcinoma, and SiHa cells for squamous cell carcinoma. The cells were cultured in DMEM medium supplemented with 10% fetal bovine serum, 2 mmol/L glutamine, 100 IU/mL penicillin, and 0.1 mg/mL streptomycin at 37°C, 5% CO_2_, and saturated humidity. Adherent cells were detached using 0.25% trypsin for passaging. Upon reaching 80%–90% confluence, cells were subcultured. During this period, medium was replaced every 2 days and cells were passaged 2-3 times per week. Cells from passages 3-10 after resuscitation were used for experiments, and the same frozen cell batch was utilized in each experiment.

Cell identification is performed using Short Tandem Repeat (STR) analysis. A suitable amount of specimen is extracted using the Microread Genomic DNA Kit to obtain DNA. The MicroreaderTM 21 ID System is utilized to amplify 20 STR loci and gender identification loci, and PCR products are detected using the ABI 3730xl Genetic Analyzer. The analysis of the detection results is conducted using GeneMapperID-X software (Applied Biosystems).

### 2.3 Animals and experiment *in vivo*

The study adhered to internationally accepted principles for laboratory animal use and care, as outlined in the US NIH guidelines (Publication #85-23, revised in 1985). Logarithmically growing HeLa cells were detached with 0.25% trypsin, terminating the digestion of the culture medium. Following low-speed centrifugation for 5 min, the supernatant was discarded. A cell suspension was prepared using Matrigel matrix glue, adjusting the cell concentration to 1 × 10^7^ cells/mL, and placed on ice. Cells were rapidly injected into the subcutaneous region of nude mice’s right buttocks using a 1 mL syringe with a No. Six needle, administering 0.1 mL of cell suspension. After 5 days of observation, tumor formation was assessed. Mice with 3.5–4.0 mm diameter tumors were considered successful models, while those with excessively large or small tumors were excluded. Experimental animals were divided into four groups: negative control group, positive control group, high-dose GEB group (10 mg/mL), and low-dose GEB group (5 mg/mL). The negative control group received 0.1 mL of normal saline every 3 days for a total of 10 administrations. The positive control group was treated with cisplatin, 5 mg/kg.bw, dissolved in normal saline, 0.1 mL/piece, administered once a week for a total of 4 weeks. The low-dose GEB group received 5 mg/mL GEB in normal saline, 0.1 mL/animal/day, with 10 administrations in total. Finally, the high-dose GEB group was treated with 10 mg/mL GEB in normal saline, 0.1 mL/animal/3 days, with a total of 10 administrations. Treatment lasted for 4 weeks, and animals were observed for a subsequent 4 weeks.

### 2.4 CCK8 assay

To assess cell viability, cells in the logarithmic growth phase were collected. Cells were added to 96-well plates at a density of 5,000 cells per well. Three replicate wells were established for each group. DMEM was added to reach a final volume of 200 µL. Cells were incubated at 37°C with 5% CO_2_. Prior to measuring the optical density, 20 µL of CCK8 solution was added to each well, followed by incubation for 2 h at 37°C in a 5% CO_2_ incubator. Absorbance was measured at 450 nm using a microplate reader, and growth curves were plotted based on absorbance values.

### 2.5 Transwell assay

The ECM culture medium was diluted with Matrigel at a ratio of 5:1, and 50 μL per well was evenly spread on the Transwell chamber and allowed to stand at room temperature for 2 h to allow the Matrigel to solidify. Cell suspensions were prepared for each group, adjusting the cell density to 2 × 10^5^ cells/mL. A total of 200 µL of the suspension was transferred to the chamber, followed by the addition of DMEM medium supplemented with 10% FBS to the lower chamber. After 24 h of incubation at 37°C, cells in the upper chamber were wiped with a wet cotton swab, fixed with anhydrous methanol, and stained with crystal violet dye. The number of stained cells in ten random fields per assay was recorded for cell migration analysis. Three replicate wells were established for each group.

### 2.6 Transmission electron microscope (TEM)

Cell samples were prepared according to standard procedures. A 2.5% glutaraldehyde and phosphoric acid buffer solution were prepared and used to fix the samples for 2 h. Samples were then dehydrated in a 4°C refrigerator. Following embedding and curing, ultrathin sections were cut using a Leica EMUC7 ultramicrotome at 70 nm thickness. The samples were double-stained with 2% uranyl acetate and lead citrate and were observed using a transmission electron microscope HT7800 operating at an acceleration voltage of 80 kV.

### 2.7 MDC staining

An autophagy staining kit (SOLARBIO, G0170) was used to detect autophagy. To prepare the MDC working solution, 9 mL of cell suspension was added to an EP tube, followed by the addition of 1 mL of MDC Stain, mixing gently. Excess liquid was removed from cell slides, and slides were washed with 1× Wash buffer. A total of 250 µL of MDC working solution was added per well, followed by incubation at room temperature in the dark for 45 min. Slides were washed with 1× Wash buffer, mounted with glycerol, and observed using a microscope. Three replicate wells were established for each group.

### 2.8 Reactive oxygen species (ros) detecting

Cell culture conditions were maintained in DMEM+10% FBS at 37°C and 5% CO_2_. Cells were washed twice with PBS, digested with trypsin, and centrifuged at 1,000 RPM for 5 min to collect the cell pellet. The cells were then resuspended in serum-free medium with a concentration of 50,000 to 100,000 cells/mL. DCFH-DA ROS fluorescence probe powder was dissolved in DMSO to a final concentration of 10 mM, then further diluted with serum-free medium at a 1:1,000 dilution ratio to obtain a final working dye concentration of 10 µM. After centrifuging the cell suspension at 1,000 RPM for 5 min and discarding the supernatant, cells were resuspended in the dye solution and incubated in the dark at 37°C for 20 min with gentle mixing every 3–5 min. Flow cytometry was used to analyze the samples, counting 10,000 cells per sample. ROS-positive cells displayed strong green fluorescence when excited at 480 nm and measured for emission near 520 nm. Three replicate wells were established for each group.

### 2.9 Detection of mitochondrial membrane potential JC-1

Cervical cancer cells were treated with 5 mg/mL GEB and 10 mg/mL GEB, respectively, for 48 h. Cells were then collected through trypsin digestion and centrifugation, pelleted, and resuspended in 500 µL of complete medium. To prepare the JC-1 dye solution, an appropriate amount of JC-1 (200×) was diluted in 8 mL ultrapure water, followed by vortexing for thorough mixing. Next, 2 mL of 5× JC-1 dye buffer was added and mixed well to create the JC-1 staining working solution. A total of 0.5 mL of JC-1 dye solution was added to each cell sample, mixed gently by inversion, and incubated at 37°C for 20 min. Meanwhile, the 5× JC-1 dye buffer was further diluted with 4 mL distilled water to prepare a 1× JC-1 dye buffer, which was placed on ice. After incubation, cells were centrifuged at 600 g and 4°C for 3–4 min, and the supernatant was discarded. Cells were then washed twice in 1× JC-1 dye buffer, as previously described, and analyzed for changes in mitochondrial membrane potential. Three replicate wells were established for each group.

### 2.10 Western blot analysis

Total cell protein was extracted, quantified according to the kit instructions, and separated using 10% SDS-PAGE gel at a constant voltage of 110 V for 80 min. The primary antibody (1:50) was dissolved in T-TBS solution and incubated at 4°C for 24 h. The internal control β-actin was dissolved in T-TBS solution at a 1:1,000 dilution. The secondary antibody was added and incubated at 37°C for 1.5 h, followed by three washes with T-TBS for 10 min each. ECL working solution (1 mL) was added to the membrane and incubated at 37°C for 1 min before being covered with a protective film. Membranes were exposed to an X-ray film in the dark, and protein bands were visualized using chemiluminescence imaging. Band intensities were quantified using ImageJ software.

### 2.11 Hematoxylin and eosin (HE) staining

Paraffin-embedded tissue sections (4 µm thickness) were prepared for H&E staining. Sections were deparaffinized and rehydrated, stained with Harris hematoxylin, rinsed with running water, and dehydrated with gradient alcohol. Samples were stained with 1% ethanol eosin, dehydrated with gradient alcohol, and excess xylene was removed before mounting with neutral resin and capturing images using a panoramic scanner.

### 2.12 Immunocytochemistry staining

Tumor slides were fixed in absolute methanol solution and blocked with 5% BSA solution at 37°C for 1 h. Slides were incubated overnight at 4 °C with primary antibodies: mouse anti-CD 90 antibody (1:200; Abcam), mouse anti-CD 105 antibody (1:200; Abcam), and mouse anti-CD 45 antibody (1:200; Abcam). The next day, slides were incubated with secondary antibody: anti-mouse FITC (1:200). Lastly, the slides were incubated with DAPI (1:1,000) and sealed with fluorescent mounting gel.

### 2.13 Statistical analysis

Statistical analysis was performed by using SPSS 22.0 software. The count data was presented as frequency (percentage) and analyzed by *χ* ^*2*^ test. The measurement data was presented as mean ± standard deviation (X±SD). Independent *t*-test was performed to analysis differences between groups while paired *t*-test was used to analysis differences within group. The grade data was tested by Mann-Whitney U test. Two-sides *p* < 0.05 was considered as statistically significant.

## 3 Results

### 3.1 GEB regulates the cell cycle of cervical cancer cells *in vitro*

**FIGURE 1:**
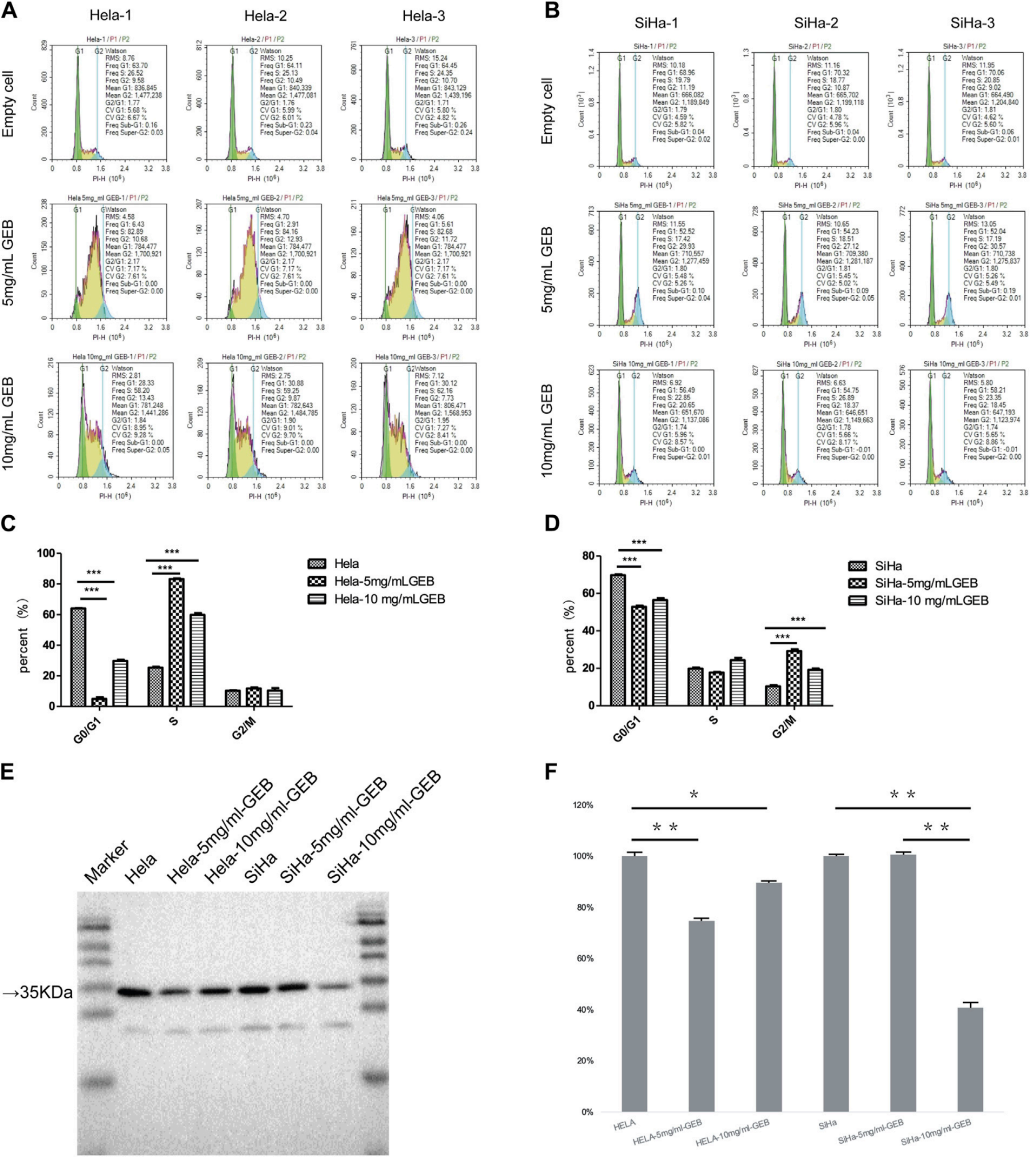
Induction of cell cycle by GEB. **(A)** Hela cell cycle distribution diagram. **(B)** SiHa cell cycle distribution diagram. **(C)** Compared with the empty cell group, the Hela-5 mg/mLGEB group showed significant differences in G0/G1 and S phases (*p* < 0.001), which were decreased by 92.22% and increased by 228.59% respectively; Compared with the empty cell group, Hela-10 mg/mLGEB group showed significant differences in G0/G1 and S phases (*p* < 0.001), which were 53.54% lower and 136.33% higher respectively. **(D)** Compared with the empty cell group, the SiHa-5 mg/mLGEB group had a statistical difference in G0/G1 and G2/M phases (*p* < 0.001), which decreased by 24.14% and increased by 181.92% respectively; Compared with the empty cell group, the SiHa-10 mg/mLGEB group had a statistical difference in G0/G1 and G2/M phases (*p* < 0.001), which decreased by 19.06% and increased by 84.91%, respectively. **(E)** Western blot results of CDK1. **(F)** Gray analysis diagram of CDK1.

We prepared GEB and used the HeLa and SiHa cell lines for *in vitro* experiments. GEB’s intervention concentrations were 5 mg/mL and 10 mg/mL. Figure 1 demonstrates that HeLa and SiHa cell lines’ G0/G1 and S phases exhibited significant differences after GEB intervention (*p* < 0.001). In HeLa cells, GEB primarily induced cell cycle arrest in the S phase (Figures 1A, C, *p* < 0.001). In SiHa cells, GEB predominantly induced cell cycle arrest in the G2/M phase (Figures 1B, D, *p* < 0.001). We predict that the CDK1 protein is approximately 34 kDa. Our experimental results indicate that protein bands were detected between Marker 15–35 KDa (Figure 1E). Grayscale analysis results suggest that 5 mg/mL and 10 mg/mL GEB concentrations can inhibit CDK1 expression compared with untreated cells (Figure 1F).

### 3.2 GEB induced apoptosis of cervical cancer cells *in vitro*

**FIGURE 2:**
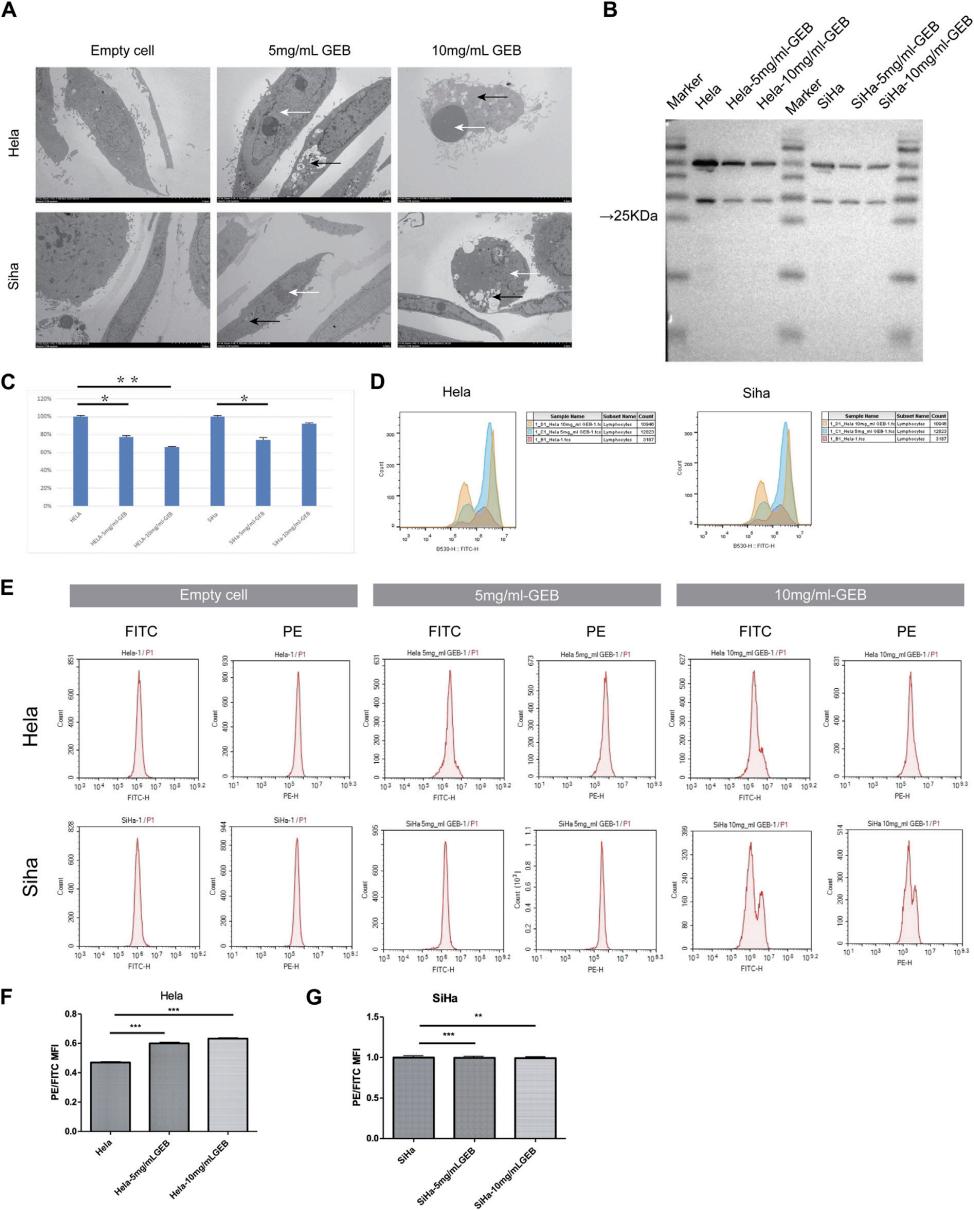
GEB induces apoptosis of cervical cancer cells *in vitro*. **(A)** The formation of autophagosome (black arrow) and apoptotic bodies (white arrow). **(B)** Western blot results of caspase3. **(C)** Gray analysis diagram of caspase3. **(D)** The ROS content of samples from Hela-5 mg/mL GEB, Hela-10 mg/mL GEB, SiHa-5 mg/mL GEB and SiHa-10 mg/mL GEB drug treatment groups increased. **(E)** Flow cytometry. **(F)** The mitochondrial membrane potential of Hela-5 mg/mL GEB and Hela-10 mg/mL GEB groups increased. **(G)** Mitochondrial membrane potential decreased slightly in SiHa-5 mg/mL GEB and SiHa-10 mg/mL GEB groups.

Given the consistent cell cycle results, electron microscopy reported the presence of apoptotic bodies (Figure 2A). Western blot results revealed that the expression of caspase3 protein in HeLa cells treated with 5 mg/mL and 10 mg/mL GEB was 77% and 66% lower, respectively (Figures 2B, C), when compared with the untreated HeLa samples. In SiHa cells treated with 5 mg/mL and 10 mg/mL GEB, the caspase3 protein expression was 74% and 92% lower, respectively (Figures 2B, C), when compared with the untreated SiHa samples. Upon GEB intervention, the caspase3 protein expression decreased, which may indicate an increase in caspase3 activation for inducing cervical cancer cell apoptosis. The ROS detection results displayed an increase in ROS content in samples treated with GEB (Figure 2D). The mitochondrial membrane potential of HeLa cells increased in the 5 mg/mL and 10 mg/mL GEB groups (Figures 2E, F), while that of SiHa cells slightly decreased in the 5 mg/mL and 10 mg/mL GEB groups (Figures 2E, G).

### 3.3 GEB promotes autophagy of cervical cancer cells *in vitro*

**FIGURE 3:**
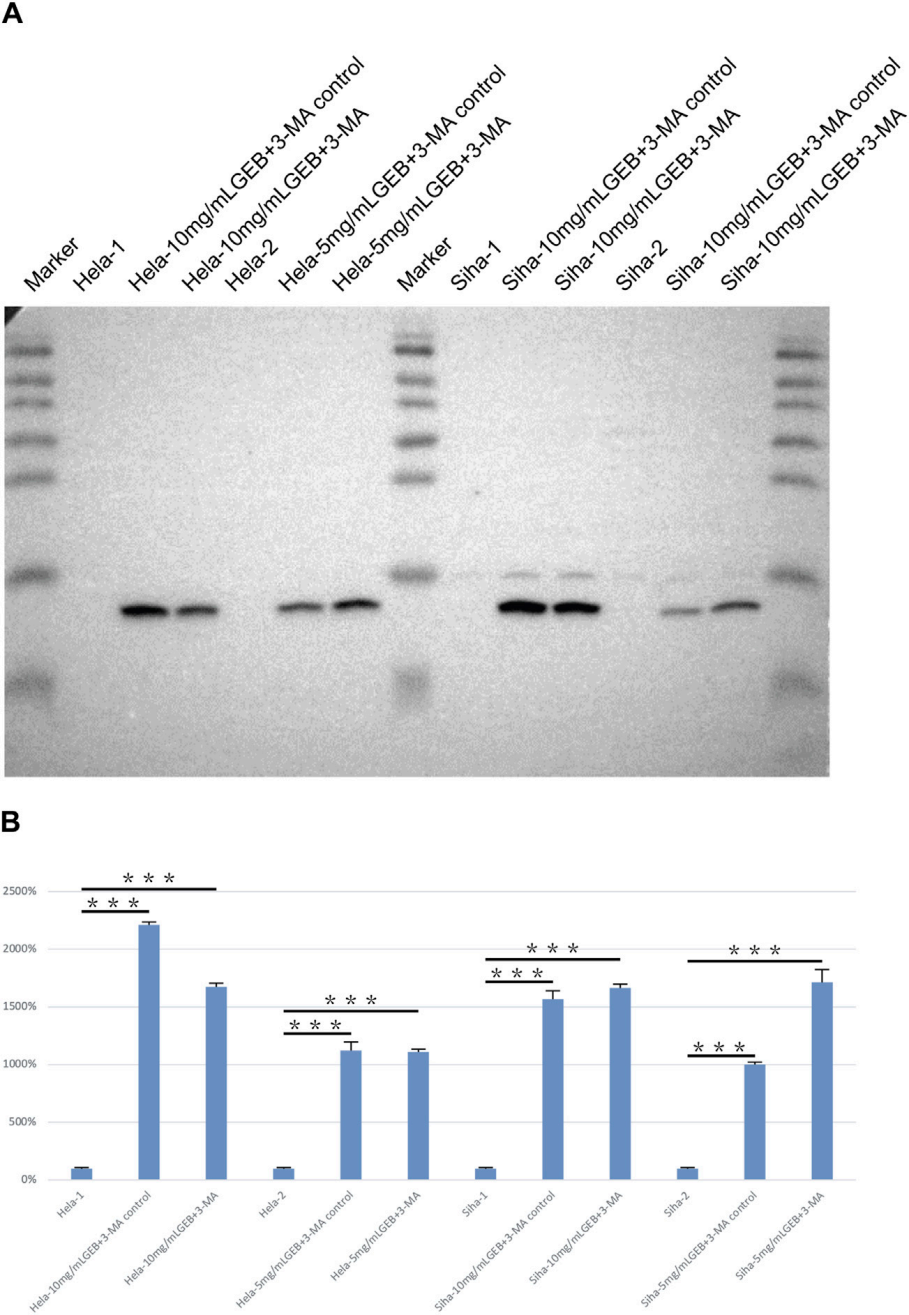
Detection of LC3A/B protein expression. **(A)** Western blot results of LC3A/B. **(B)** Gray analysis diagram of LC3A/B.

Under the electron microscope, GEB was observed to enhance autophagosome (Figure 2A, black arrow) and apoptotic bodies formation (white arrow) in HeLa and SiHa cells. We treated the HeLa and SiHa cells with GEB and 3-MA drugs of varying concentrations for 48 h. The CCK-8 method quantified cell proliferation in different concentration groups, examining the effects of GEB and 3-MA drugs on HeLa cell proliferation. The CCK-8 results suggested that no significant changes occurred in proliferative capacity, and specific IC50 could not be analyzed. We predict that the LC3A/B protein is approximately 14–16 KDa. Western blot results detected protein bands between Marker 10–15 kDa (Figure 3A). After conducting the gray-scale analysis, our results showed that GEB significantly increased LC3A/B protein expression in HeLa and SiHa cells (Figure 3B). In comparison with untreated HeLa samples, the LC3A/B protein expression in the treated samples increased substantially (Figure 3B). A similar increase was observed in the treated SiHa samples when compared with untreated SiHa samples (Figure 3B).

### 3.4 GEB inhibits the migration, invasion and proliferation of cervical cancer cells *in vitro*

**FIGURE 4:**
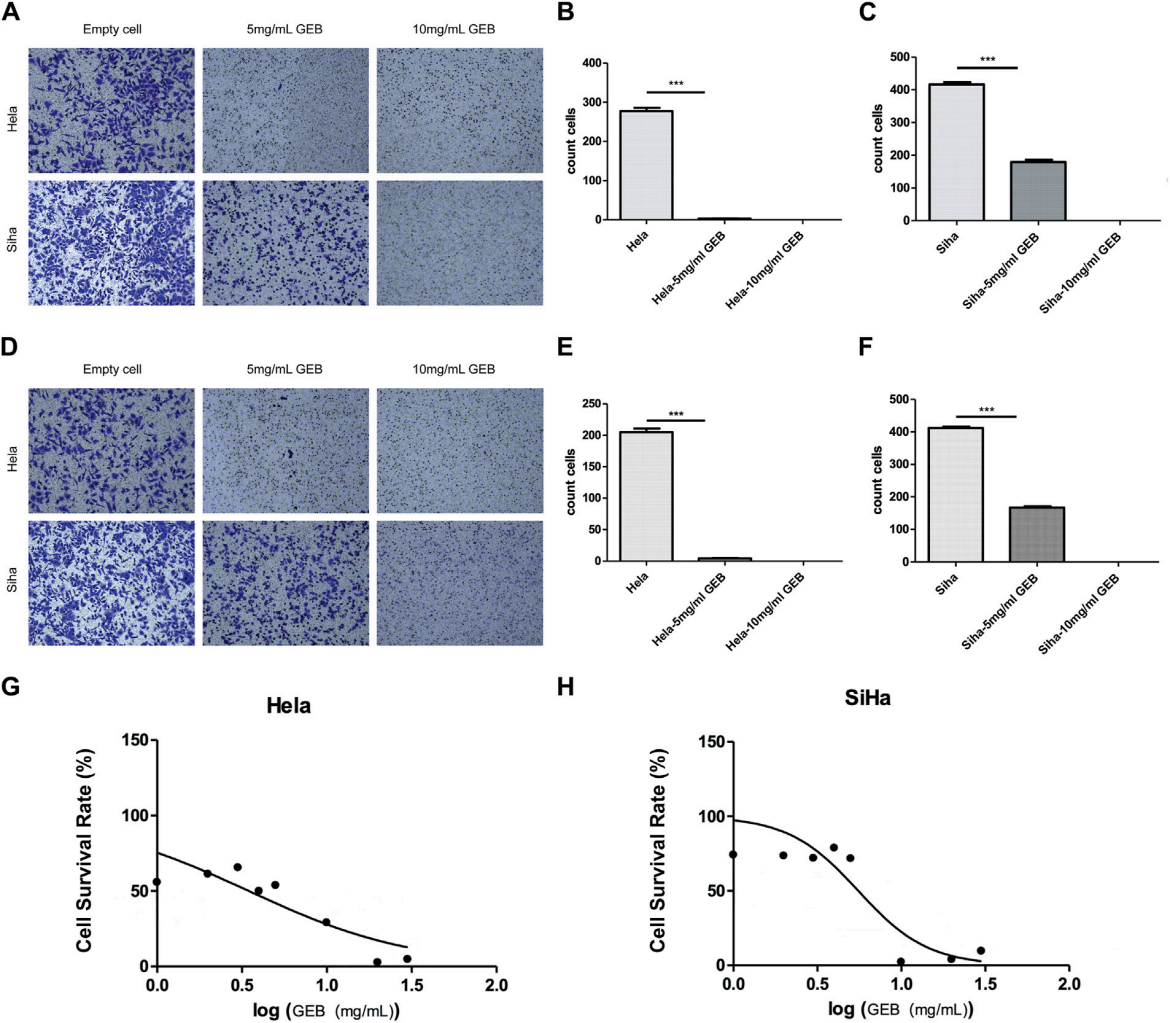
GEB inhibits the migration, invasion, and proliferation of cervical cancer cells *in vitro*. **(A)** Effect of GEB on the migration ability of Hela and Siha cells. **(B)** Statistical analysis results of GEB drugs on the migration ability of Hela cells. **(C)** Statistical analysis results of GEB drugs on the migration ability of Siha cells. **(D)** Effect of GEB on the invasion ability of Hela and Siha cells. **(E)** Statistical analysis results of GEB drugs on the invasion ability of Hela cells. **(F)** Statistical analysis results of GEB drugs on the invasion ability of Siha cells. **(G)** Effects of GEB on the proliferation of Hela cells. **(H)** Effects of GEB on the proliferation of SiHa cells.

GEB inhibits the migration, invasion, and proliferation of cervical cancer cells *in vitro*. The Transwell assay results demonstrated that GEB inhibited the migration and invasion of cervical cancer cells, with a more significant inhibitory effect observed at the 10 mg/mL GEB concentration (Figures 4A–F, *p* < 0.001). The CCK-8 assay evaluated GEB’s effect on tumor proliferation, revealing that the IC50 values for HeLa and SiHa cells corresponded to GEB drug concentrations of 3.472 mg/mL and 5.538 mg/mL, respectively (Figures 4G, H). These findings indicate that GEB has a noteworthy impact on tumor proliferation, and theoretically, 10 mg/mL GEB would produce a higher inhibitory effect.

### 3.5 GEB inhibits the growth and angiogenesis of cervical cancer cells *in vivo*

**FIGURE 5:**
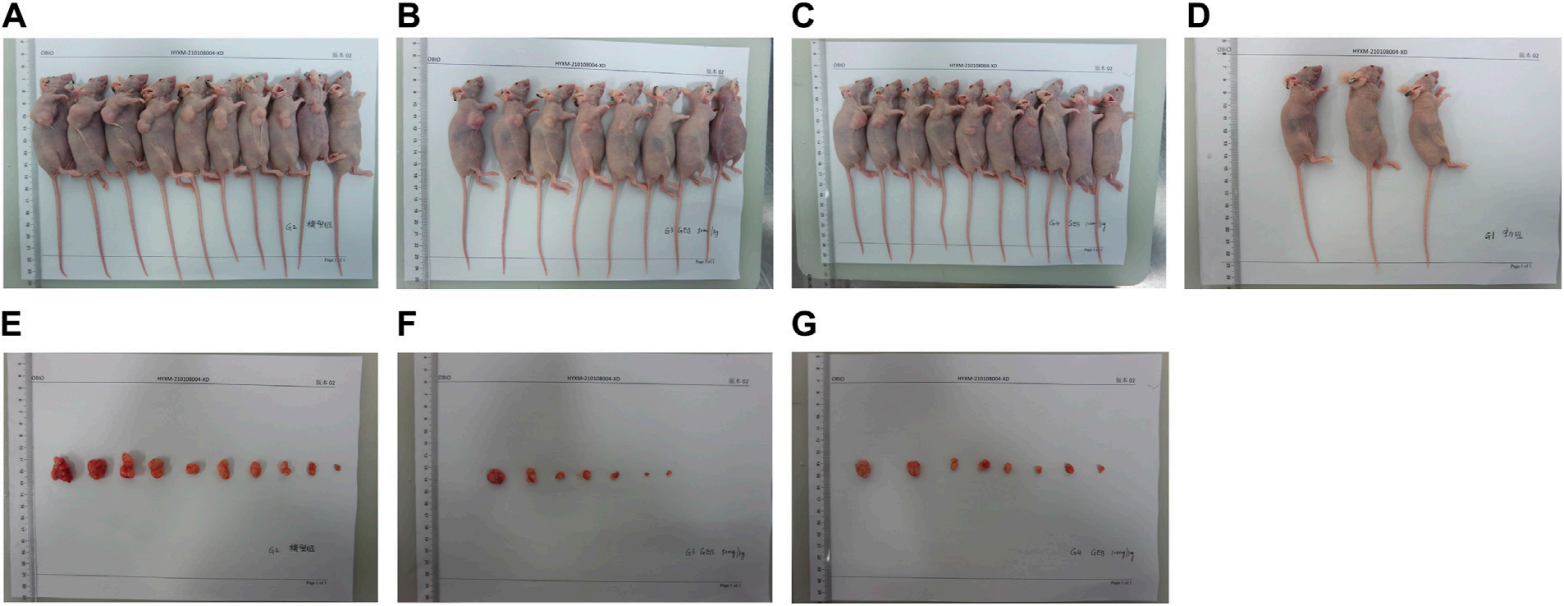
Experimental animal model. **(A)** Tumor nude mice of G2 group *in vivo*. **(B)** Tumor nude mice of G3 group *in vivo*. **(C)** Tumor nude mice of G4 group *in vivo*. **(D)** Nude mice in G1 group. **(E)** Tumor nude mice of G2 group *in vitro*. **(F)** Tumor nude mice of G3 group *in vitro*. **(G)** Tumor nude mice of G4 group *in vitro*.

**FIGURE 6:**
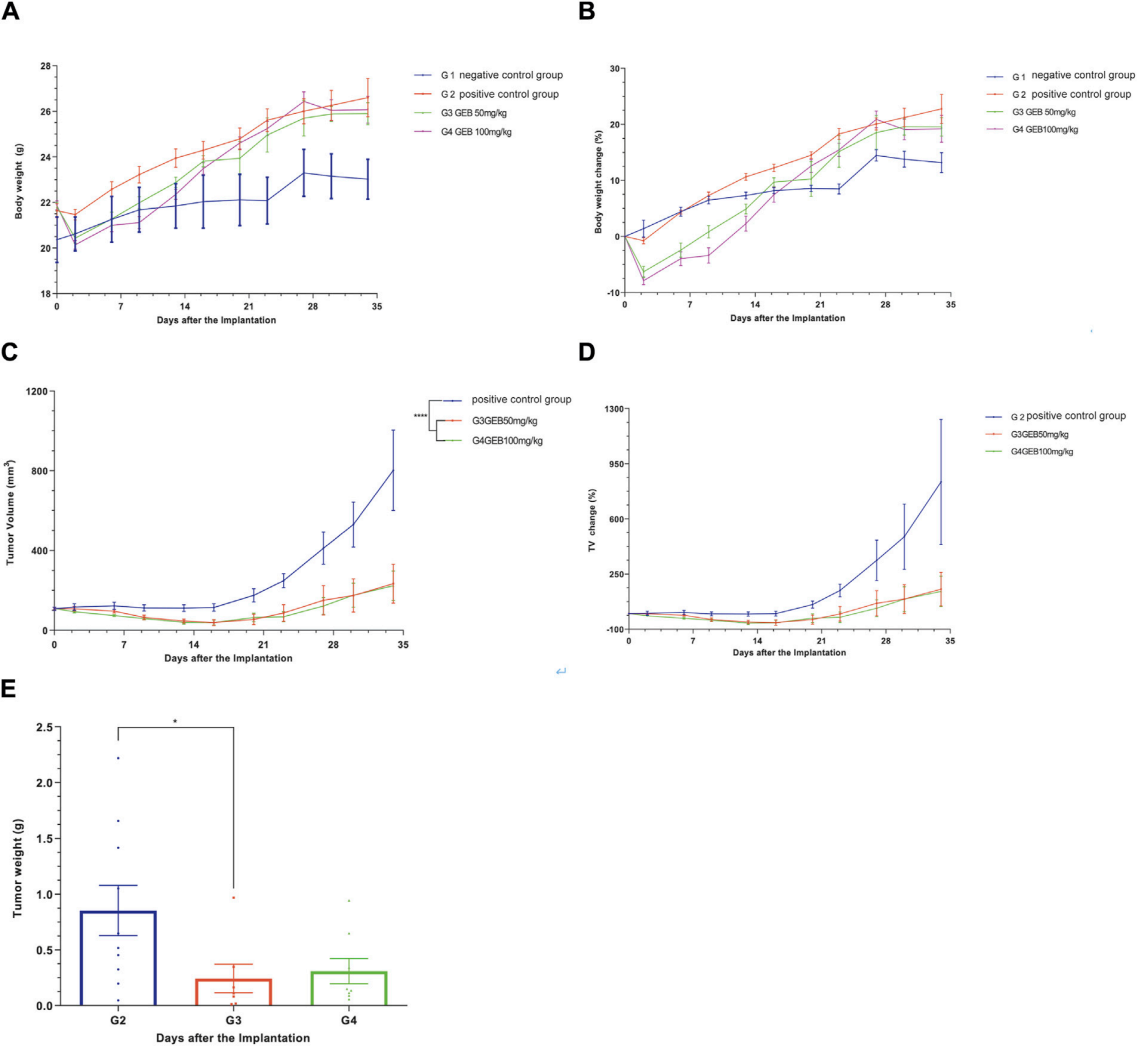
Efficacy evaluation of subcutaneous tumorigenesis in *vitvo*. **(A)** Body weight of nude mice. **(B)** Body weight changes in nude mice. **(C)** Tumor volume in nude mice. **(D)** Changes in tumor volume in nude mice. **(E)** Tumor weight of nude mice.

**FIGURE 7:**
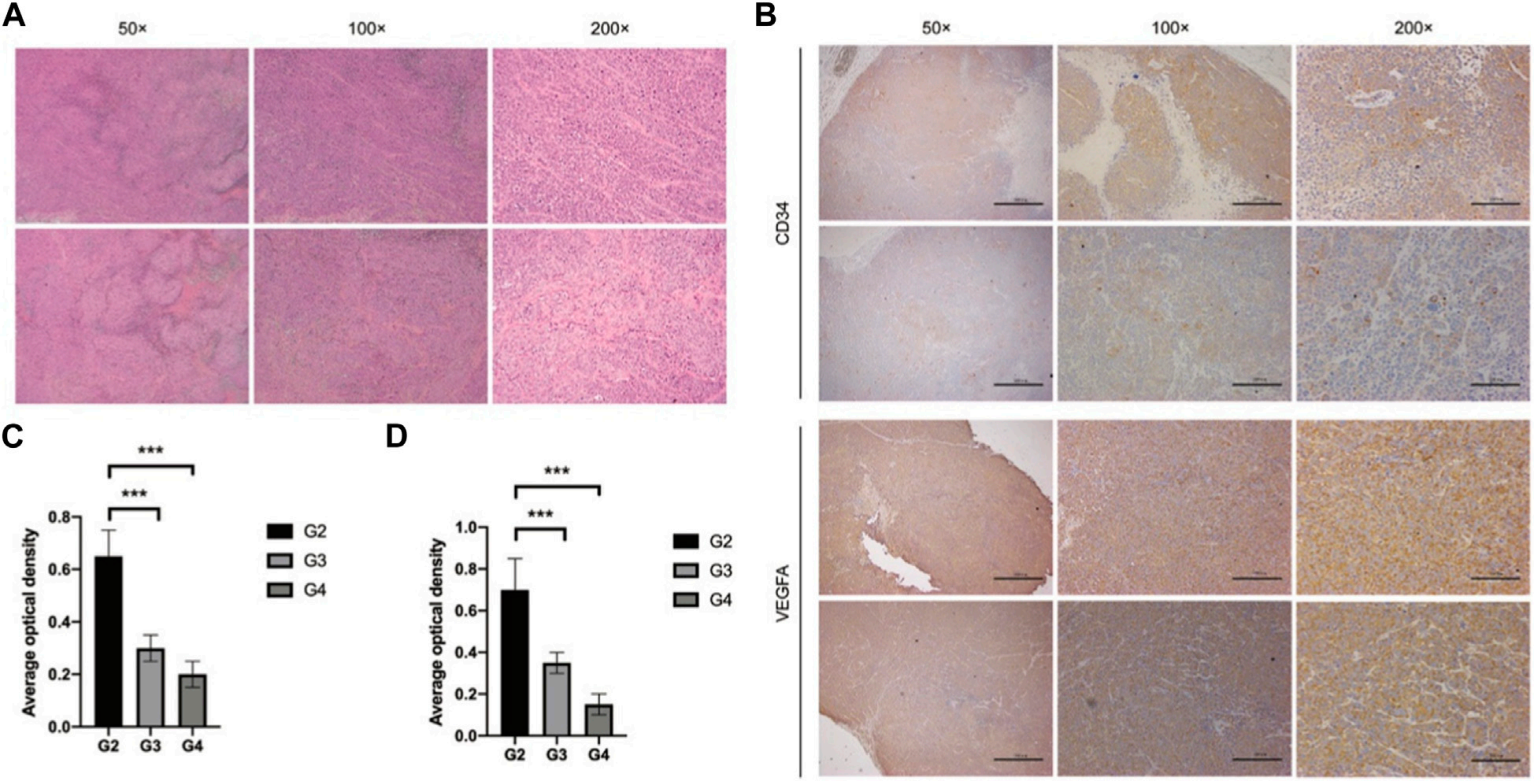
Tumor tissue detection. **(A)** HE staining. **(B)** Immunohistochemical staining. **(C)** Average optical density of CD34. **(D)** Average optical density of VEGF.

In our *in vivo* investigation, we established a cervical cancer xenograft model in nude mice using HeLa cells (Figure 5). The experimental animals were divided into the negative control group (G1), positive control group (G2), low-dose GEB group (G3), and high-dose GEB group (G4). After establishing the model, the weight of nude mice in groups G2, G3, and G4 significantly increased (Figures 6A, B). In comparison with G2, the tumor volume and weight in G3 and G4 decreased considerably (Figures 6C–E, *p* < 0.01, *p* < 0.0001). HE staining demonstrated that GEB intervention considerably inhibited tumor angiogenesis (Figure 7A) and suppressed the expression of CD34 and VEGF in tumor tissue (Figures 7B–D).

## 4 Discussion

Despite significant advancements in global medical standards, early screening for gynecological tumors, such as cervical cancer (CC), remains a widespread issue worldwide (Bedell et al., 2020). While early screening has led to a decrease in the number of patients with advanced tumors (Rajaram and Gupta, 2021), CC continues to be a leading cause of cancer-related deaths among women globally (Sung et al., 2021). The standard treatment methods for women with CC in most countries include surgical treatment, radiotherapy, and chemotherapy (Marnitz et al., 2020; Bhatla et al., 2021). However, surgical treatment combined with lymph node dissection has limited effectiveness in patients with advanced CC due to metastasis. Complementary treatments such as synchronous radiation therapy and chemotherapy can result in damage to normal cells and are not always successful due to drug resistance and other issues, leading to poor prognoses for some patients (Gadducci and Cosio, 2020; Wendel and Leath, 2020). Despite the availability of mature treatment options, the prognosis for advanced CC patients remains grim, underscoring the need to develop new, safe, and effective treatments for CC.

An increasing body of evidence supports the anti-tumor properties of Traditional Chinese Medicines (TCMs) and their active components, including Taohong Siwu Decoction (Jiang et al., 2021), Danshen (Li et al., 2021), Shenling Baizhu (Feng et al., 2020), and others. The anti-tumor effects of numerous TCM compounds and their active ingredients have been demonstrated in various gynecological tumors, such as cervical cancer (Banik et al., 2022), endometrial cancer (Tsai et al., 2021), breast cancer (Yang et al., 2021), and ovarian cancer (Chan et al., 2020). GEB, a formula specifically formulated by our research team for cervical cancer, comprises D. versipellis, D. dasycarps, and A. dahurica. In this investigation, we isolated the core components of GEB through a refinement process and explored their anti-tumor effects in cervical cancer. The experimental results revealed substantial and comprehensive anti-tumor activity of GEB against cervical cancer. GEB inhibited the proliferation of both HeLa and SiHa cells, leading to cell cycle arrest in the G2/M phase. The formation of autophagic and apoptotic bodies served as crucial indicators of decreased tumor cell biological activity, which were significantly promoted by GEB intervention. The expression of autophagy and apoptosis-related proteins further corroborated the enhancement of autophagy and apoptosis. Additionally, the migration and invasion assays of HeLa and SiHa cell lines demonstrated that GEB suppressed the biological activity of cervical cancer cells. Moreover, the measurement of ROS content and mitochondrial membrane potential indicated that GEB might induce apoptosis by boosting mitochondrial oxidative stress and increasing mitochondrial membrane permeability. Consequently, the significant and wide-ranging anti-tumor effects of GEB against cervical cancer cells were established.

The *in vivo* experiments have effectively developed a nude mouse model of cervical cancer transplantation using HeLa cells, and have confirmed the substantial inhibitory impact of GEB on tumor growth and tumor angiogenesis. It is worth noting that the anti-tumor activity of GEB shows a significant correlation with its concentration, with 10 mg/mL demonstrating greater effectiveness compared to 5 mg/mL. Combining *in vitro* and *in vivo* experimental data, we can conclude that GEB is an effective inhibitor of CC and possesses significant and extensive anti-tumor activity.

The active ingredients in TCMs form the basis for their therapeutic effects. We analyzed the source of GEB’s anti-tumor activity, focusing on PTOX, imperatorin, isoimperatorin, and *A. dahurica* alkaloids as the four active molecules with tumor cell activity inhibition. PTOX, one of GEB’s core components, exhibits potent microtubule or DNA damaging capabilities and provides a wide-ranging and efficient anti-tumor effect. The US FDA approved podophyllin drugs, including etoposide (VP-16) (Wu et al., 2011), teniposide (VM-26) (Tepper and Studzinski, 1992), and etoposide phosphate (Etopophos), for treating small cell lung cancer in the 1980s and 1990s. Since then, various podophyllotoxin antineoplastic drugs have been developed for addressing lung cancer, leukemia, lymphatic cancer, breast cancer, testicular cancer glioma, and other human malignancies, often with good results. Imperatorin and isoimperatorin belong to the 6,7-furacoumarins, and coumarins exhibit higher application value and more biological activities. Numerous coumarins are present in Angelica dahurica. Okuyama (Okuyama et al., 1990) and others isolated six coumarin components from the anti-tumor active part of Angelica dahurica, with imperatorin and isoimperatorin displaying the strongest anti-tumor effects. Lv (Lv et al., 2021) and others concluded that imperatorin can induce autophagy and G0/G1 phase arrest of human osteosarcoma cells both *in vitro* and *in vivo* via the PTEN-PI3K-AKT-mTOR/p21 signaling pathway. Kim (Kim et al., 2022) and others determined that isoimperatorin regulates NF in colorectal cancer cells and liver cancer cells, downregulating epithelial-mesenchymal transition via κB signaling and CXCR4 expression. Recent studies corroborated that plant alkaloids have anti-tumor effects, and their anti-tumor mechanisms are associated with interfering with tumor cell cycles, inducing tumor cell apoptosis, inhibiting tumor angiogenesis, and multidrug resistance (Chen et al., 2021; Wang et al., 2021). Additionally, *A. dahurica* contains abundant Angelica alkaloids and water-soluble polysaccharides. Dong (Dong et al., 2021) and others demonstrated that Angelica dahurica polysaccharide (ADP) exhibited significant anti-tumor activity in H22 tumor-bearing mice. Hwangbo (Hwangbo et al., 2020) and others extracted *A. dahurica* alkaloids and verified their inhibitory effect on the growth and metastasis of murine melanoma B16F10 cells.

**FIGURE 8:**
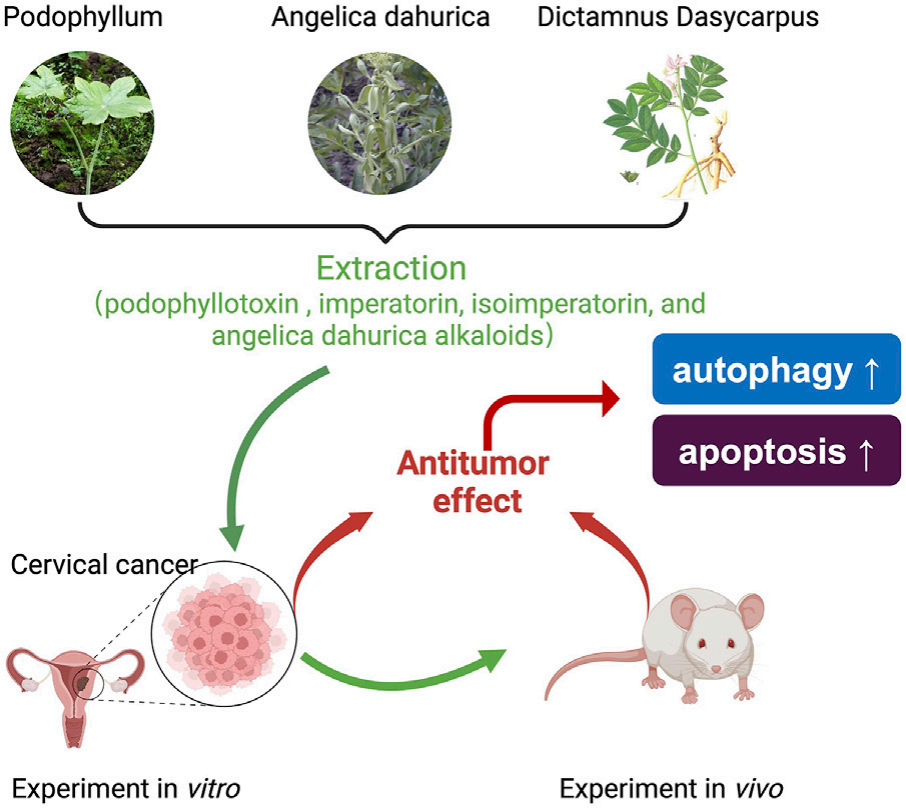
Schematic diagram of proposed mechanism. GEB, a complex formulation, originates from the traditional Chinese medicine of the Miao ethnicity and is composed of podophyllotoxin (PTOX), imperatorin, isoimperatorin, and *A. dahurica alkaloids*. GEB has demonstrated a potent antitumor efficacy against cervical cancer, both in laboratory experiments and in living organisms, in a manner dependent on the concentration, through the modulation of autophagy and apoptosis in cancer cells.

Through the comprehensive analysis of GEB’s anti-tumor active components, we found that these components have broad anti-tumor effects and action types (Figure 8). This suggests that GEB may not be limited to treating cervical cancer but could also provide significant activity against other tumor types. However, this conclusion requires further verification. Importantly, these core active ingredients in GEB can be synthesized industrially, paving the way for standardized production and clinical application of GEB in the future.

This study has some limitations. The compatibility of compound Chinese medicine should adhere to specified proportions. However, we did not measure the content of specific active molecules after extracting effective ingredients but instead used mixed ingredient concentrations for *in vivo* and *in vitro* experiments. Although this study confirmed the concentration dependence of GEB’s anti-tumor effect, its optimal dominant concentration might necessitate further exploration with refined concentration gradients in future research. Additionally, we are currently investigating the application effect of GEB in CC patients, and this study lacks data on GEB’s impact on CC patient prognosis. Our research group plans to address these limitations in future work.

## 5 Conclusion

In this study, we demonstrated the significant anti-tumor effects of GEB on cervical cancer through *in vivo* and *in vitro* experiments. GEB regulates the proliferation, metastasis, migration, and invasion of tumor cells by modulating autophagy and apoptosis in a concentration-dependent manner. Additionally, GEB may exhibit substantial inhibitory effects on other tumor types, warranting further investigation.

## Data Availability

The original contributions presented in the study are included in the article/Supplementary Material, further inquiries can be directed to the corresponding author.

## Abbreviations

CC, Cervical cancer; FIGO, Federation International of Gynecology and Obstetrics; GEB, GuiErBai; PTOX, podophyllotoxin; ADP, *A. dahurica* polysaccharide.

## References

[B1] Banik K. Khatoon E. Harsha C. Rana V. Parama D. Thakur K. K. (2022). Wogonin and its analogs for the prevention and treatment of cancer: a systematic review. Phytother. Res. 36 (5), 1854–1883. 10.1002/ptr.7386 35102626

[B2] Barret J. M. Kruczynski A. Vispé S. Annereau J. P. Brel V. Guminski Y. (2008). F14512, a potent antitumor agent targeting topoisomerase II vectored into cancer cells via the polyamine transport system. Cancer. Res. 68 (23), 9845–9853. 10.1158/0008-5472.CAN-08-2748 19047165

[B3] Bedell S. L. Goldstein L. S. Goldstein A. R. Goldstein A. T. (2020). Cervical cancer screening: past, present, and future. Sex. Med. Rev. 8 (1), 28–37. 10.1016/j.sxmr.2019.09.005 31791846

[B4] Bhatla N. Aoki D. Sharma D. N. Sankaranarayanan R. (2021). Cancer of the cervix uteri: 2021 update. Int. J. Gynaecol. Obstet. 155 (Suppl. 1), 28–44. 10.1002/ijgo.13865 34669203 PMC9298213

[B5] Bhattacharjee R. Dey T. Kumar L. Kar S. Sarkar R. Ghorai M. (2022). Cellular landscaping of cisplatin resistance in cervical cancer. Biomed. Pharmacother. 153, 113345. 10.1016/j.biopha.2022.113345 35810692

[B6] Chan D. W. Yung M. M. Chan Y. S. Xuan Y. Yang H. Xu D. (2020). MAP30 protein from Momordica charantia is therapeutic and has synergic activity with cisplatin against ovarian cancer *in vivo* by altering metabolism and inducing ferroptosis. Pharmacol. Res. 161, 105157. 10.1016/j.phrs.2020.105157 32814169

[B7] Chen M. C. Pan S. L. Shi Q. Xiao Z. Lee K. H. Li T. K. (2012). QS-ZYX-1-61 induces apoptosis through topoisomerase II in human non-small-cell lung cancer A549 cells. Cancer. Sci. 103 (1), 80–87. 10.1111/j.1349-7006.2011.02103.x 21920000 PMC11164173

[B8] Chen M. H. Gu Y. Y. Zhang A. L. Sze D. M. Mo S. L. May B. H. (2021). Biological effects and mechanisms of matrine and other constituents of Sophora flavescens in colorectal cancer. Pharmacol. Res. 171, 105778. 10.1016/j.phrs.2021.105778 34298110

[B9] De Nola R. Loizzi V. Cicinelli E. Cormio G. (2021). Dynamic crosstalk within the tumor microenvironment of uterine cervical carcinoma: baseline network, iatrogenic alterations, and translational implications. Crit. Rev. Oncol. Hematol. 162, 103343. 10.1016/j.critrevonc.2021.103343 33930531

[B10] Dong X. D. Liu Y. N. Zhao Y. Liu A. J. Ji H. Y. Yu J. (2021). The structural characteristics of an acid-soluble polysaccharide from Grifola frondosa and its antitumor effects on H22-bearing mice. Int. J. Biol. Macromol. 193 (Pt A), 1288–1298. 10.1016/j.ijbiomac.2020.05.054 32437807

[B11] Feng Z. Feng Z. Han J. Cheng W. Su B. Mo J. (2020). Antinociceptive effects of shenling Baizhu through PI3K-Akt-mTOR signaling pathway in a mouse model of bone metastasis with small-cell lung cancer. Evid. Based. Complement. Altern. Med. 2020, 4121483. 10.1155/2020/4121483 PMC7327581 32655659

[B12] Ferrall L. Lin K. Y. Roden R. B. S. Hung C. F. Wu T. C. (2021). Cervical cancer immunotherapy: facts and hopes. Clin. Cancer. Res. 27 (18), 4953–4973. 10.1158/1078-0432.CCR-20-2833 33888488 PMC8448896

[B13] Floberg J. M. Zhang J. Muhammad N. DeWees T. A. Inkman M. Chen K. (2021). Standardized uptake value for 18F-fluorodeoxyglucose is a marker of inflammatory state and immune infiltrate in cervical cancer. Clin. Cancer. Res. 27 (15), 4245–4255. 10.1158/1078-0432.CCR-20-4450 33820781 PMC8338789

[B14] Gadducci A. Cosio S. (2020). Neoadjuvant chemotherapy in locally advanced cervical cancer: review of the literature and perspectives of clinical research. Anticancer. Res. 40 (9), 4819–4828. 10.21873/anticanres.14485 32878770

[B15] Gamage C. D. B. Park S. Y. Yang Y. Zhou R. Taş İ. Bae W. K. (2019). Deoxypodophyllotoxin exerts anti-cancer effects on colorectal cancer cells through induction of apoptosis and suppression of tumorigenesis. Int. J. Mol. Sci. 20 (11), 2612. 10.3390/ijms20112612 31141929 PMC6601030

[B16] Gao L. Zhong L. Zhang J. Zhang M. Zeng Y. Li L. (2021). Water as a probe to understand the traditional Chinese medicine extraction process with near infrared spectroscopy: a case of Danshen (Salvia miltiorrhiza Bge) extraction process. Spectrochim. Acta. A. Mol. Biomol. Spectrosc. 244, 118854. 10.1016/j.saa.2020.118854 32920500

[B17] Guo Q. Jiang E. (2021). Recent advances in the application of podophyllotoxin derivatives to fight against multidrug-resistant cancer cells. Curr. Top. Med. Chem. 21 (19), 1712–1724. 10.2174/1568026621666210113163327 33441065

[B18] He W. Q. Li C. (2021). Recent global burden of cervical cancer incidence and mortality, predictors, and temporal trends. Gynecol. Oncol. 163 (3), 583–592. 10.1016/j.ygyno.2021.10.075 34688503

[B19] Hwangbo H. Choi E. O. Kim M. Y. Kwon D. H. Ji S. Y. Lee H. (2020). Suppression of tumor growth and metastasis by ethanol extract of Angelica dahurica Radix in murine melanoma B16F10 cells. Biosci. Trends. 14 (1), 23–34. 10.5582/bst.2019.01230 32092745

[B20] Jiang H. Li M. Du K. Ma C. Cheng Y. Wang S. (2021). Traditional Chinese medicine for adjuvant treatment of breast cancer: Taohong Siwu decoction. Chin. Med. 6 (1), 129. 10.1186/s13020-021-00539-7 PMC8638166 34857023

[B21] Kalam M. A. Malik A. H. Ganie A. H. Butt T. A. (2021). Medicinal importance of papra (podophyllum hexandrum royle) in unani system of medicine. J. Complement. Integr. Med. 18 (3), 485–490. 10.1515/jcim-2020-0178 33544520

[B22] Kamal A. Laxman N. Ramesh G. (2000). Facile and efficient one-pot synthesis of 4beta-arylaminopodophyllotoxins: synthesis of DNA topoisomerase II inhibitors (NPF and W-68). Bioorg Med. Chem. Lett. 10 (18), 2059–2062. 10.1016/s0960-894x(00)00407-8 10999470

[B23] Keilholz U. Rohde L. Mehlitz P. Knoedler M. Schmittel A. Kümmerlen V. (2017). First-in-man dose escalation and pharmacokinetic study of CAP7.1, a novel prodrug of etoposide, in adults with refractory solid tumours. Eur. J. Cancer. 80, 14–25. 10.1016/j.ejca.2017.03.032 28531881

[B24] Khalaf K. Hana D. Chou J. T. Singh C. Mackiewicz A. Kaczmarek M. (2021). Aspects of the tumor microenvironment involved in immune resistance and drug resistance. Front. Immunol. 12, 656364. 10.3389/fimmu.2021.656364 34122412 PMC8190405

[B25] Kim N. Y. Jung Y. Y. Yang M. H. Um J. Y. Sethi G. Ahn K. S. (2022). Isoimperatorin down-regulates epithelial mesenchymal transition through modulating NF-κB signaling and CXCR4 expression in colorectal and hepatocellular carcinoma cells. Cell. Signal. 99, 110433. 10.1016/j.cellsig.2022.110433 35934221

[B26] Kuo M. L. Shen S. C. Yang C. H. Chuang S. E. Cheng A. L. Huang T. S. (1998). Bcl-2 prevents topoisomerase II inhibitor GL331-induced apoptosis is mediated by down-regulation of poly(ADP-ribose)polymerase activity. Oncogene 17 (17), 2225–2234. 10.1038/sj.onc.1202133 9811453

[B27] Li H. Gao C. Liu C. Liu L. Zhuang J. Yang J. (2021). A review of the biological activity and pharmacology of cryptotanshinone, an important active constituent in Danshen. Biomed. Pharmacother. 137, 111332. 10.1016/j.biopha.2021.111332 33548911

[B28] Lv M. Xu Q. Zhang B. Yang Z. Xie J. Guo J. (2021). Imperatorin induces autophagy and G0/G1 phase arrest via PTEN-PI3K-AKT-mTOR/p21 signaling pathway in human osteosarcoma cells *in vitro* and *in vivo* . Cancer. Cell. Int. 21 (1), 689. 10.1186/s12935-021-02397-7 34923996 PMC8684670

[B29] Maleš Ž. Drvar D. L. Duka I. Žužul K. (2019). Application of medicinal plants in several dermatovenerological entities. Acta. Pharm. 69 (4), 525–531. 10.2478/acph-2019-0045 31639095

[B30] Mao X. Xu J. Wang W. Liang C. Hua J. Liu J. (2021). Crosstalk between cancer-associated fibroblasts and immune cells in the tumor microenvironment: new findings and future perspectives. Mol. Cancer. 20 (1), 131. 10.1186/s12943-021-01428-1 34635121 PMC8504100

[B31] Marnitz S. Tsunoda A. T. Martus P. Vieira M. Affonso Junior R. J. Nunes J. (2020). Surgical versus clinical staging prior to primary chemoradiation in patients with cervical cancer FIGO stages IIB-IVA: oncologic results of a prospective randomized international multicenter (Uterus-11) intergroup study. Int. J. Gynecol. Cancer. 30 (12), 1855–1861. 10.1136/ijgc-2020-001973 33293284 PMC7788482

[B32] Mayadev J. S. Ke G. Mahantshetty U. Pereira M. D. Tarnawski R. Toita T. (2021). Global challenges of radiotherapy for the treatment of locally advanced cervical cancer. Int. J. Gynecol. Cancer 32 (3), 436–445. 10.1136/ijgc-2021-003001 PMC8921593 35256434

[B33] Merzouki A. Buschmann M. D. Jean M. Young R. S. Liao S. Gal S. (2012). Adva-27a, a novel podophyllotoxin derivative found to be effective against multidrug resistant human cancer cells. Anticancer. Res. 32 (10), 4423–4432. PMID: 23060568. 23060568

[B34] Nasser M. I. Zhu S. Hu H. Huang H. Guo M. Zhu P. (2019). Effects of imperatorin in the cardiovascular system and cancer. Biomed. Pharmacother. 120, 109401. 10.1016/j.biopha.2019.109401 31622950

[B35] Okuyama T. Takata M. Nishino H. Nishino A. Takayasu J. Iwashima A. (1990). Studies on the antitumor-promoting activity of naturally occurring substances. II. Inhibition of tumor-promoter-enhanced phospholipid metabolism by umbelliferous materials. Chem. Pharm. Bull. (Tokyo) 38 (4), 1084–1086. 10.1248/cpb.38.1084 2379282

[B36] Pagani O. Zucchetti M. Sessa C. de Jong J. D'Incalci M. De Fusco M. (1996). Clinical and pharmacokinetic study of oral NK611, a new podophyllotoxin derivative. Cancer. Chemother. Pharmacol. 38 (6), 541–547. 10.1007/s002800050524 8823496

[B37] Pal A. Das S. Basu S. Kundu R. (2022). Apoptotic and autophagic death union by Thuja occidentalis homeopathic drug in cervical cancer cells with thujone as the bioactive principle. J. Integr. Med. 20 (5), 463–472. 10.1016/j.joim.2022.06.004 35752587

[B38] Perrin D. van Hille B. Barret J. M. Kruczynski A. Etiévant C. Imbert T. (2020). F 11782, a novel epipodophylloid non-intercalating dual catalytic inhibitor of topoisomerases I and II with an original mechanism of action. Biochem. Pharmacol. 59 (7), 807–819. 10.1016/s0006-2952(99)00382-2 10718339

[B39] Rajaram S. Gupta B. (2021). Screening for cervical cancer: choices and dilemmas. Indian. J. Med. Res. 154 (2), 210–220. 10.4103/ijmr.IJMR_857_20 34854432 PMC9131755

[B40] Rashmi R. Jayachandran K. Zhang J. Menon V. Muhammad N. Zahner M. (2020). Glutaminase inhibitors induce thiol-mediated oxidative stress and radiosensitization in treatment-resistant cervical cancers. Mol. Cancer. Ther. 19 (12), 2465–2475. 10.1158/1535-7163.MCT-20-0271 33087507 PMC8208465

[B41] Ruiz F. J. Inkman M. Rashmi R. Muhammad N. Gabriel N. Miller C. A. (2021). HPV transcript expression affects cervical cancer response to chemoradiation. JCI. insight. 6 (16), e138734. 10.1172/jci.insight.138734 34255749 PMC8409981

[B42] Ruiz F. J. Sundaresan A. Zhang J. Pedamallu C. S. Halle M. K. Srinivasasainagendra V. (2021). Genomic characterization and therapeutic targeting of HPV undetected cervical carcinomas. Cancers (Basel) 13 (18), 4551. 10.3390/cancers13184551 34572780 PMC8467954

[B43] Singh D. Vignat J. Lorenzoni V. Eslahi M. Ginsburg O. Lauby-Secretan B. (2023). Global estimates of incidence and mortality of cervical cancer in 2020: a baseline analysis of the WHO Global Cervical Cancer Elimination Initiative. Lancet. Glob. Health 11 (2), e197–e206. 10.1016/S2214-109X(22)00501-0 36528031 PMC9848409

[B44] Sung H. Ferlay J. Siegel R. L. Laversanne M. Soerjomataram I. Jemal A. (2021). Global cancer statistics 2020: GLOBOCAN estimates of incidence and mortality worldwide for 36 cancers in 185 countries. Ca. Cancer. J. Clin. 71 (3), 209–249. 10.3322/caac.21660 33538338

[B45] Tepper C. G. Studzinski G. P. (1992). Teniposide induces nuclear but not mitochondrial DNA degradation. Cancer. Res. 52 (12), 3384–3390. 1596897

[B46] Tsai Y. T. Kuo P. H. Kuo H. P. Hsu C. Y. Lee Y. J. Kuo C. L. (2021). Ganoderma tsugae suppresses the proliferation of endometrial carcinoma cells via Akt signaling pathway. Environ. Toxicol. 36 (3), 320–327. 10.1002/tox.23037 33044769

[B47] Wang K. Chen Q. Shao Y. Yin S. Liu C. Liu Y. (2021). Anticancer activities of TCM and their active components against tumor metastasis. Biomed. Pharmacother. 133, 111044. 10.1016/j.biopha.2020.111044 33378952

[B48] Wang L. B. Wang D. N. Wu L. G. Cao J. Tian J. H. Liu R. (2021). Homoharringtonine inhibited breast cancer cells growth via miR-18a-3p/AKT/mTOR signaling pathway. Int. J. Biol. Sci. 17 (4), 995–1009. 10.7150/ijbs.44907 33867824 PMC8040299

[B49] Wang S. Fu J. L. Hao H. F. Jiao Y. N. Li P. P. Han S. Y. (2021). Metabolic reprogramming by traditional Chinese medicine and its role in effective cancer therapy. Pharmacol. Res. 170, 105728. 10.1016/j.phrs.2021.105728 34119622

[B50] Wang X. Y. Ding X. Yuan Y. F. Zheng L. Y. Cao Y. Zhu Z. Y. (2018). Comprehensive two-dimensional APTES-decorated MCF7-cell membrane chromatographic system for characterizing potential anti-breast-cancer components from Yuanhu-Baizhi herbal medicine pair. J. Food. Drug. Anal. 26 (2), 823–833. 10.1016/j.jfda.2017.11.010 29567254 PMC9322241

[B51] Wendel Naumann R. Leath C. A. 3rd. (2020). Advances in immunotherapy for cervical cancer. Curr. Opin. Oncol. 32 (5), 481–487. 10.1097/CCO.0000000000000663 32740092 PMC7748319

[B52] Wollina U. (2003). Er:YAG laser followed by topical podophyllotoxin for hard-to-treat palmoplantar warts. J. Cosmet. Laser. Ther. 5 (1), 35–37. PMID: 12745597. 10.1080/14764170305500 12745597

[B53] Wu C. C. Li T. K. Farh L. Lin L. Y. Lin T. S. Yu Y. J. (2011). Structural basis of type II topoisomerase inhibition by the anticancer drug etoposide. Science 333 (6041), 459–462. 10.1126/science.1204117 21778401

[B54] Yang Z. Zhang Q. Yu L. Zhu J. Cao Y. Gao X. (2021). The signaling pathways and targets of traditional Chinese medicine and natural medicine in triple-negative breast cancer. J. Ethnopharmacol. 264, 113249. 10.1016/j.jep.2020.113249 32810619

